# Patterns of Change in Dietary Habits and Physical Activity during Lockdown in Spain Due to the COVID-19 Pandemic

**DOI:** 10.3390/nu13020300

**Published:** 2021-01-21

**Authors:** Carmen Pérez-Rodrigo, Marta Gianzo Citores, Gotzone Hervás Bárbara, Fátima Ruiz-Litago, Luis Casis Sáenz, Victoria Arija, Ana M. López-Sobaler, Emilio Martínez de Victoria, Rosa M. Ortega, Teresa Partearroyo, Joan Quiles-Izquierdo, Lourdes Ribas-Barba, Amelia Rodríguez-Martín, Gemma Salvador Castell, Josep A. Tur, Gregorio Varela-Moreiras, Lluis Serra-Majem, Javier Aranceta-Bartrina

**Affiliations:** 1 Department of Physiology, Faculty of Medicine, University of the Basque Country (UPV/EHU), 48940 Leioa, Biscay, Spain; fatima.ruiz@ehu.eus (F.R.-L.); luis.casis@ehu.eus (L.C.S.); jaranceta@unav.es (J.A.-B.); 2 Spanish Society of Community Nutrition (SENC), 08029 Barcelona, Spain; marta.gianzo@ehu.eus (M.G.C.); gotzone.hervas@gmail.com (G.H.B.); victoria.arija@urv.cat (V.A.); asobaler@ucm.es (A.M.L.-S.); emiliom@ugr.es (E.M.d.V.); rortega@ucm.es (R.M.O.); t.partearroyo@ceu.es (T.P.); quiles_joa@gva.es (J.Q.-I.); fin@fin.pcb.ub.es (L.R.-B.); amelia.rodriguez@uca.es (A.R.-M.); gemma.salvador@gencat.cat (G.S.C.); pep.tur@uib.es (J.A.T.); gvarela@ceu.es (G.V.-M.); lluis.serra@ulpgc.es (L.S.-M.); 3 Nutrition and Mental Health Research Group (NUTRISAM), Institut d’Investigació Sanitària Pere Virgili (IISPV), Universitat Rovira i Virgili, 43201 Reus, Tarragona, Spain; 4 Nutrición Sin Fronteras, 08029 Barcelona, Spain; 5 Departamento de Nutrición y Ciencia de los Alimentos, Facultad de Farmacia, Universidad Complutense de Madrid, 28040 Madrid, Spain; 6 Institute of Nutrition and Food Sciences, University of Granada, 18010 Granada, Spain; 7 Fundación Iberoamericana de Nutrición, FINUT, 18016 Armilla Granada, Spain; 8 Departamento de Ciencias Farmacéuticas y de la Salud, Facultad de Farmacia, Universidad San Pablo-CEU, CEU Universities, Urbanización Montepríncipe, 28925 Alcorcón, Madrid, Spain; 9 Conselleria de Sanidad Universal y Salud Pública, Generalitat Valenciana, 46020 Valencia, Spain; 10 CIBERESP (Consortium for Biomedical Research in Epidemiology and Public Health), Carlos III Health Institute, 28029 Madrid, Spain; 11 CIBEROBN, Biomedical Research Networking Center for Physiopathology of Obesity and Nutrition, Carlos III Health Institute, 28029 Madrid, Spain; 12 Fundación para la Investigación Nutricional (FIN), 08029 Barcelona, Spain; 13 Departamento de Biomedicina, Biotecnología y Salud Pública, Facultad de Enfermería y Fisioterapia Universidad de Cádiz, 11009 Cádiz, Spain; 14 Departament de Salut, Generalitat de Catalunya, 08005 Barcelona, Spain; 15 Research Group on Community Nutrition & Oxidative Stress, University of the Balearic Islands, 07122 Palma de Mallorca, Spain; 16 Health Research Institute of the Balearic Islands (IdISBa), 07120 Palma de Mallorca, Spain; 17 Spanish Nutrition Foundation (FEN), 28010 Madrid, Spain; 18 Research Institute of Biomedical and Health Sciences (IUIBS), University of Las Palmas de Gran Canaria, and Complejo Hospitalario Universitario Insular—Materno Infantil (CHUIMI), Canarian Health Service, 35016 Las Palmas de Gran Canaria, Spain; 19 Department of Food Sciences and Physiology, University of Navarra, Pamplona, 31009 Navarra, Spain

**Keywords:** COVID-19 pandemic, lifestyles, food consumption, physical activity, supplements, confinement, patterns

## Abstract

Background: Lockdown due to COVID-19 influenced food habits and lifestyles with potential negative health impact. This study aims to identify patterns of change in eating habits and physical activity during COVID-19 lockdown in Spain and to identify associations with sociodemographic factors and usual habits. Methods: This cross-sectional study included 1155 adults recruited online to answer a 10-section questionnaire. The protocol assessed usual diet by means of a semi-quantitative food frequency questionnaire, usual physical activity (PA) and supplement use, dietary changes, sedentary time, PA, exposure to sunlight, sleep quality, and smoking during confinement. Patterns of dietary change were identified by factor analysis. Factor scores were included in cluster analysis together with change in PA. Results: Six patterns of dietary change were identified that together with PA changes during lockdown defined three clusters of lifestyle change: a cluster less active, a more active cluster, and a third cluster as active as usual. People who were usually less active were more likely to be classified in the cluster that increased physical activity in confinement. Scores of the Healthy Mediterranean-Style dietary pattern were higher in this group. **Conclusions:** Different patterns of change in lifestyles in confinement suggest the need to tailor support and advice to different population groups.

## 1. Introduction

The outbreak of the global pandemic caused by SARS-CoV-2 (Severe Acute Respiratory Syndrome Coronavirus 2), the causal agent of COVID-19, rapidly expanding from the first focus notified in Wuhan (China), has caused to date (1 December 2020) more than 63,890,000 people affected and over 1,480,000 deaths worldwide [1] across the five continents.

Hygienic means and physical distancing, including lockdown, have been the most effective strategies to contain the progression and severity of the pandemic [2] to date, despite intense research activity in search of effective and safe vaccines, along with specific treatments, with limited results so far.

Spain has been one of the European countries most affected by the pandemic. On 14 March 2020, the Government declared a state of alarm in the country and established lockdown, with movement restriction, physical distancing, and isolation for citizens (RD463/2020). The most restrictive confinement measures in Spain lasted 49 days (from 15 March to 2 May 2020), which was followed by four phases of de-escalation progressively allowing greater outdoor mobility, return to on-site work, and leisure within the framework of the so-called “new normal” [3]. During that period, except for health care personnel and those in essential services, all citizens should remain at home most of the time. Lockdown norms allowed people to go out just to buy food and strictly access basic supplies in nearby food stores and supermarkets, but not for a walk or exercising. Even all children should remain at home 24 h a day. Lockdown conditions in Spain were among the most restrictive in Europe.

Many citizens suffered the stress generated by the pandemic, with alarming numbers of affected people and the uncertainty of the socioeconomic consequences of lockdown [4]. There is evidence that anxiety and stress influence behaviors and food choices, in addition to affecting hunger and satiety sensation [5]. Lockdown and social isolation have been associated to psychological and emotional disturbances, changes in mood, sleep disorders, changes in eating habits, and a decrease in exposure to sunlight, in addition to favoring a sedentary lifestyle with less physical activity (PA) [6].

In addition, lockdown also influenced food-shopping practices despite the food supply being assured at all times, although with some constraints [7]. A number of authors have reported changes in food habits during confinement in several countries [8,9,10,11] and in different age groups [9,12,13]. Changes in PA and quality of sleep associated with confinement have been reported as well [14,15,16,17]. All those changes in lifestyles can influence the evolution of chronic diseases and risk factors in many people [12,13,16,18,19] with a negative health impact.

On the other hand, despite there being no solid evidence of specific nutrients that could benefit the prevention or treatment of COVID-19, nutritional modulation of the immune system could play an important role across lifespan, which is especially relevant in older adults due to the impact of COVID-19 on this age group [20,21]. Omega-3 polyunsaturated fatty acids and probiotics have been related to anti-inflammatory response and increased resistance to upper respiratory infections [20,22]. Some research suggest that dietary supplementation with vitamins A, B, C, and D as well as minerals such as selenium, zinc, and iron, omega-3, and melatonin could play a role as co-adjuvants in the treatment of COVID-19 patients and for the prevention of upper respiratory tract infections, although evidence is limited [22,23,24].

In April 2020, when the most restrictive conditions were in force in Spain, market research companies, the Ministry of Agriculture, Fisheries and Food, and other sources published information about changes in purchases of food and other goods. We conducted a cross-sectional online self-reported survey on a convenience sample with the aim of exploring changes in eating habits and related practices, physical activity, sleep, and other habits during lockdown for the pandemic [25]. Some reported changes were reduced consumption of chocolate (25.8%), sugary drinks (32.8%), and distilled alcoholic beverages (44.2%), as well as processed meats (35.5%), lamb or rabbit meat (32%), or pizza (32.6%). In addition, 27% reported increased consumption of fruit, eggs (25.4%), legumes (22.5%), vegetables (21%), and fish (20%). However, changes in consumption varied with gender, age group, cohabitation during lockdown, and quality of usual diet [25].

Experts predict the pandemic to last for over 18 months, with waves of increased cases potentially requiring severe movement restrictions that may affect people’s lives, undermine citizens’ health, and limit the capacity of overwhelmed health care services, with many people having to remain under quarantine isolation [26]. A better understanding of changes in health-related behaviors, such as diet and physical activity, in this context would provide important information to design targeted health promotion actions and tailored advice for the population.

In recent years, analysis of dietary patterns has gained attention in food consumption studies, using a variety of methodological approaches [27]. Data-driven methods explore the similarities between different food options in population groups. Furthermore, such methods have been used to gain insight into the potential combination of different health-related behaviors. Cluster analyses include several techniques aimed at grouping together individuals sharing a number of features or similar lifestyles. This classification allows for a better understanding of the influences of behaviors and lifestyles as well as the potential cumulative effects of an unhealthy combination of those factors [28].

Hence, in this analysis, we sought to identify patterns of change in eating habits, physical activity, and other health-related behaviors during COVID-19 lockdown in Spain and to identify associations with sociodemographic factors and usual habits.

## 2. Materials and Methods

The data analyzed derive from a cross-sectional study conducted in a convenience sample of people aged 18 years and over who were recruited online, through social media, the website and newsletter of the Spanish Society of Community Nutrition (SENC) following a snowball procedure. Due to the nature of the sampling methods, it was not possible to calculate specific response rates. However, in the first level of dissemination, 1200 individuals were invited to participate. Across all recruitment strategies, 1155 potential participants initially began the survey, of which 104 did not complete at least 75% of the content and 15 people resided in other countries, so they were excluded from this analysis (*n* = 1036; 301 men, 735 women).

### 2.1. Measures

#### 2.1.1. Sociodemographic Information

The questionnaire consisted of 10 sections. Regarding general sociodemographic information, the variables were gender and age group (<25 years; 25–34 years; 35–44 years; 45–54 years; 55–64 years; 65 or more years). Other items focused on specific information regarding circumstances during lockdown, such as size of locality (<1000 inhabitants; 1000–4999 inhabitants; 5000–19,999 inhabitants; 20,000–99,999 inhabitants; 100,000–299,999 inhabitants; 300,000 or more inhabitants), region, usual home (Yes/No), cohabitation during lockdown (living alone; living with family; living with partner; living with relatives, friends, or other people). In addition, there was a question about sunlight hours in the home and outdoor living space during confinement.

#### 2.1.2. Food Habits

The protocol included a previously used and validated semi-quantitative short food frequency questionnaire (FFQ) about usual food and beverage consumption, considering the last six months as a reference period. It consisted of 24 items with answer options: never; once a month or less; 2–3 times a month; once weekly; 2–3 times weekly; 4–5 times a week; every day, once; every day, several times. The short FFQ intended to capture the overall profile of usual diet.

An additional item inquired about the type of fat used for cooking and seasoning food preparations. The food groups considered are described as Supplementary Materials. Based on the FFQ data about usual food consumption, a score was computed to assess the degree of adequacy to recommendations in the Dietary Guidelines of SENC [29] (total score 0 to 15), which were subsequently categorized into quartiles.

The instrument was validated, and it aimed to be useful for measuring adherence to Spanish Dietary Guidelines (DG) by comparing it with the data from a validated 210-item quantitative FFQ in the context of the ENPE (Estudio Nutricional de la Población Española) study [30], which is a cross-sectional study conducted on a random sample of adults aged 3 years and over (2015–2016). Participants in the validation study (adults aged 18 years and older) completed the full-length quantitative FFQ in a face-to-face interview, and two weeks later, they completed the short questionnaire. Absolute agreement of individual component scoring between the short FFQ and the full-length FFQ was determined with kappa statistics. Moderate concordance (k = 0.41–0.60) was found for 13.3%, good concordance (k = 0.61–0.80) was found for 26.7%, and excellent concordance (k = 0.81–1.00) was found for 60% of the components of the score of adherence to Spanish Dietary Guidelines, respectively.

Both Pearson correlation coefficient (r = 0.85; *p* < 0.001) between the DG adherence score derived from the short FFQ and DG adherence score calculated based on the full-length FFQ, and the intra-class correlation coefficient (ICC) (ICC = 0.84; *p* < 0.001) showed that the short FFQ had an adequate ability to rank participants by the DG adherence score.

Another section asked about changes in consumption of the same groups of foods and beverages during the confinement period. Cross-sectional studies provide snapshot observations. Therefore, we asked to report about food consumption “before” and “currently during” the COVID-19 pandemic confinement. To self-report consumption during social isolation, in order to avoid survey fatigue [31], instead of asking people to report again frequency of consumption, we used a Likert-type scale to self-rate consumption for each food item, with the following answer options: much less, less, the same, more, and much more compared to the usual reported consumption before the pandemic. Internal consistency of this section of the questionnaire was assessed using Cronbach’s alpha. Cronbach’s alpha = 0.81 and a small cohort error variance of 0.31 showed the strong inter-relatedness of the 24 food items, ensuring validity [32].

A specific section queried about the consumption of vitamin and mineral supplements, as well as dietary supplements during lockdown, frequency of consumption, and type of supplement.

#### 2.1.3. Physical Activity and Sedentary Time

The current physical activity recommendations state that adults should practice 150–300 min a week of moderate-intensity activity [33]. To achieve that, adults should undertake at least 30 min of moderate-intensity activity on five or more days of the week. Milton et al. [34] developed and tested a single-item measure to assess physical activity suitable for use in a wide range of settings.

In the validation study, they observed strong agreement between the categorical variables (kappa = 0.76, confidence interval 0.69 to 0.82) and strong reproducibility of the data (pooled correlation coefficient = 0.82). Concurrent validity comparing the single question with Global Physical Activity Questionnaire (GPAQ) and the Active People Survey (APS) demonstrated fair to moderate correlation: GPAQ (r = 0.52) and APS total physical activity score (r = 0.59).

The questionnaire inquired about usual practice of moderate to vigorous PA for at least 30 min, with answer options: never; less than one day a week, two days a week; 3–4 days per week; and five or more days per week. Another item asked about practice of moderate to vigorous PA (at least 30 min) during lockdown.

A third question inquired about the time dedicated to sedentary activities daily during confinement, with response options less than 1 h; between 1 and <3 h; between 3 and <5 h; between 5 and <7 h; between 7 and <9 h; between 9 and <10 h and 10; or more hours.

#### 2.1.4. Other Practices and Lifestyles

The last section of the questionnaire asked about the frequency of exposure to sunlight through a window, at a balcony, terrace, or garden for at least 10 min during confinement, with response options ranging from never to five or more days a week. Likewise, an item inquired about usual smoking habits and another item inquired about changes in this habit during lockdown. Information on sleep quality during confinement was collected with nine statements, and Likert-type responses ranged from “I completely disagree” (−2) to “I totally agree” (2).

### 2.2. Procedure

Google forms application was used for data collection, with the anonymized option activated, so that prior registration or identification by email was not required. The survey took approximately 15 min to complete. No access internet protocol (IP) or any other data that could allow identifying participants remained registered. Responses were collected five weeks after the start of lockdown, between 21 April and 8 May 2020 (weeks 6–8 of confinement), before the start of the de-escalation phase in Spain.

### 2.3. Ethics

Participants were informed about the purpose and objectives of the research, all the data provided was anonymized, so that no participant could be identified or contacted in any case, and participation was voluntary. They were also advised that they could abandon the questionnaire at any time and, if they eventually decided to leave the form, their answers would not be saved unless they clicked to send the form at the end. Participants who accepted to continue and participate in the survey were asked to be honest in their responses.

The name, center, and contact email of a member of the research group was provided for any query or additional information request. Research was conducted in accordance with the Helsinki declaration for human studies of the World Medical Association. Strict respect for the confidentiality of the information according to Organic Law 15/1999, of December 13, of protection of personal data in all the processes of collection and treatment of the information obtained and to Organic Law 3/2018, of December 5, on the Protection of Personal Data and guarantee of digital rights.

### 2.4. Data Analysis

#### Patterns of Change in Food Consumption and Physical Activity

To analyze changes in food consumption during lockdown, a score was assigned for each food group: –2, much less consumption; –1, less consumption; 0, the same; 1, more consumption; and 2, much more consumption. We included these variables in exploratory factor analysis to identify underlying patterns of change in eating habits during confinement (*n* = 998 with valid response for all the variables).

We used Bartlett’s test of sphericity and the Kaiser–Meyer–Olkin (KMO) measure of sampling adequacy to verify the appropriateness of factor analysis and adopted a KMO value > 0.60 to assess the degree of intercorrelations between variables. Factors were also orthogonally rotated (varimax option) to enhance the difference between loadings, which allowed easier interpretability. Factors were retained based on factor eigenvalue > 1.1, identification of a break point in the scree plot, the proportion of variance explained, and factor interpretability. The strength and direction of the associations between patterns and food groups were described through a rotated factor loading matrix. Food groups with factor loadings > 0.30 were retained in the patterns identified. The factor score for each pattern was constructed by summing scores of the component food items weighted by the factor loading. A high factor score for a given pattern indicated higher scores in change of intake of the foods constituting that food factor and thus a higher intake; a low score indicated a lower intake of those foods.

Following this procedure, we identified six patterns of change in food consumption. Subsequently, the factor scores of the six factors, along with change in PA practice (*n* = 1035 with valid response) as a categorical variable (less −1; the same 0 or more +1), were used as inputs in two-stage cluster analysis to identify patterns of change in lifestyles, using log-likelihood distances. Several possible cluster solutions were identified and compared. The final 3-cluster solution was selected based on interpretability and the percent of the study population in each cluster.

The sociodemographic characteristics of participants classified in the clusters, as well as consumption of dietary supplements during lockdown, sedentary lifestyle, sleep disturbances, and smoking habits (*n* = 997) were compared using χ^2^ tests and analysis of variance. Multinomial logistic regression analysis was used to explore the association between sociodemographic factors, degree of adherence to dietary guidelines in the usual diet, usual PA, sedentary time, exposure to sunlight, and sleep disturbances during confinement, with cluster of change in lifestyles during lockdown as a dependent variable (reference category: cluster with no changes in physical activity). Following a steps-forward procedure, *p* < 0.1 as criterion of permanence, the final model included usual physical activity, degree of adherence to dietary guidelines in the usual diet, and exposure to sunlight in confinement, adjusted for age and gender. The odds ratio (OR) and its 95% confidence interval were estimated. All data analysis was conducted using SPSS statistical package (v24.0, IBM corp., Armonk, NY, USA).

## 3. Results

### 3.1. Characteristics of Participants

Women comprised 70.9% of the participants in this study, 28.3% were under 35 years of age. Some 89% reported they spent lockdown in their usual home, with their family (59.3%) or partner (25.2%), 89.8% spent lockdown in a house with windows that allowed sunlight to enter, and 44.1% had a terrace where they could go out during confinement.

Table 1 describes sociodemographic characteristics and usual lifestyles of participants. Some 14.7% declared they were usual smokers, and 86.1% stated they usually practiced 30-min sessions of moderate physical exercise at least twice or more times per week. Mean diet quality score according to the degree of adherence to the dietary guidelines was 8.79 ± 2.21.

### 3.2. Consumption of Nutritional and Dietary Supplements

**Figure 1:**
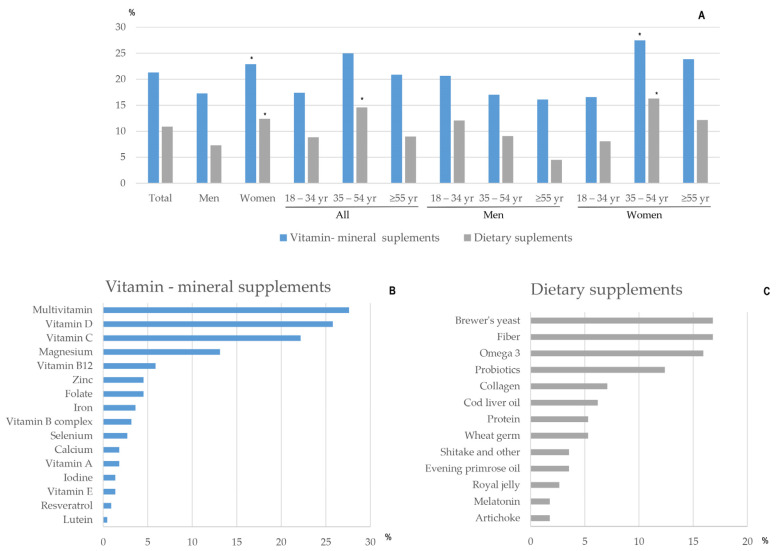
Consumption of vitamin–mineral supplements and dietary supplements during confinement in Spain: (**A**) Percentage of consumers by gender and age; (**B**) Most frequently consumed vitamin and mineral supplements; (**C**) Most frequently consumed dietary supplements. * *p* < 0.05.

At least 21.3% of participants declared consuming vitamin and mineral supplements during confinement, and there was a higher proportion in women and in people aged 35 to 54 years (Figure 1A); nine people (4.1% of consumers) reported they already consumed supplements before confinement. The supplements most frequently used were combinations of multivitamins, minerals, and trace elements (27%) (Figure 1B), which was followed by vitamin D (25.8%) and vitamin C (22.2%) in variable doses, and 10.9% reported consuming dietary supplements and herbal products. The most frequently consumed dietary supplements were brewer’s yeast (16.8%), fiber (16.8%), omega-3 polyunsaturated fatty acids (15.9%), and probiotics (12.4%) (Figure 1C).

### 3.3. Patterns of Change in Food Consumption

**Figure 2:**
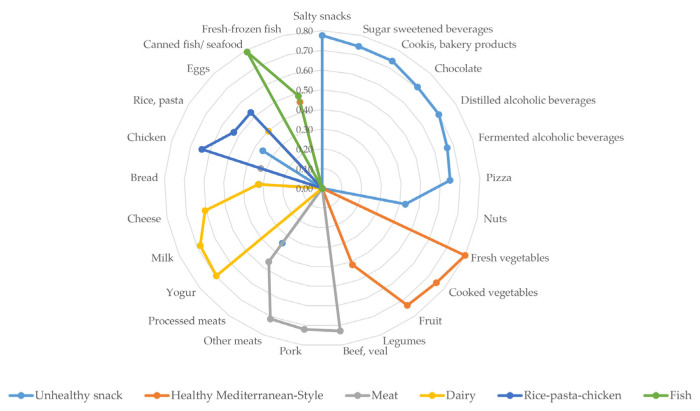
Patterns of change in eating habits during confinement in Spain. Factor scores of patterns identified by factor analysis, principal components. Coefficients < 0.30 have been deleted.

Six patterns of change in food consumption during confinement were identified, which explain 53.8% of the variance. Figure 2 shows factor scores of the matrix of rotated components. A pattern was characterized by shifts toward increased consumption of alcoholic and sugary beverages, salty snacks, cookies, bakery products, chocolate, and pizza (Unhealthy snack foods and beverage pattern). The Healthy Mediterranean-Style dietary pattern showed changes toward higher consumption of fresh and cooked vegetables, fruits, legumes, and fish. A Meat pattern described changes toward greater consumption of different kinds of meat. The Dairy pattern was characterized by higher consumption of dairy products and, to a lesser extent, eggs. The fifth pattern described a higher consumption of chicken, rice, pasta, eggs and, to a lesser extent, legumes (Rice–pasta–chicken pattern). The sixth pattern showed higher consumption of fresh, frozen, and canned fish (Fish pattern).

### 3.4. Patterns of Change in Lifestyles during Confinement

**Figure 3:**
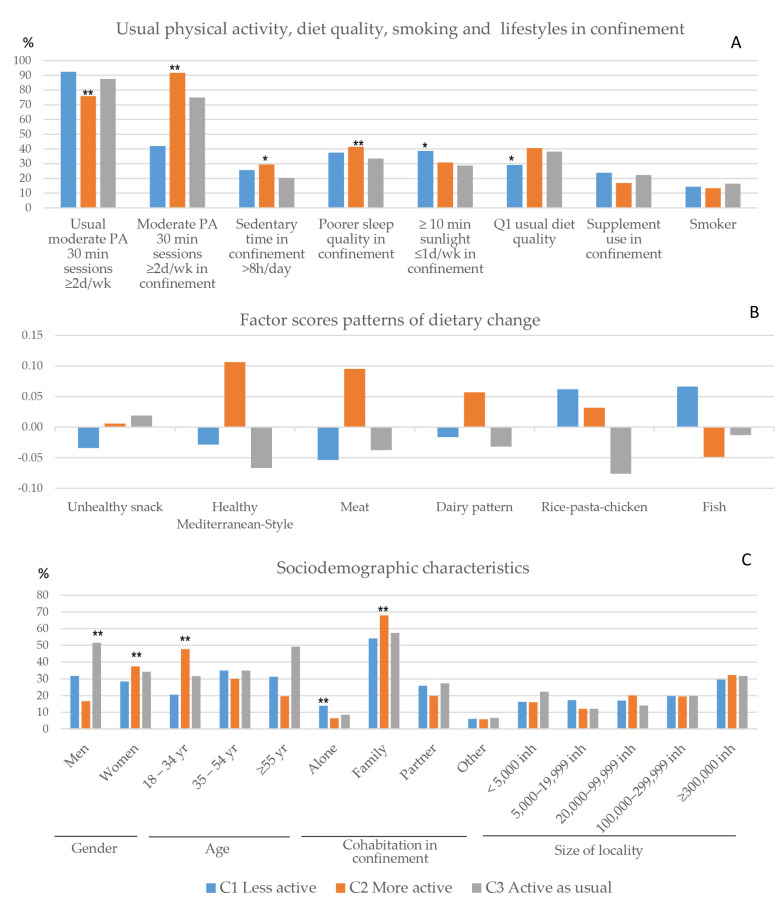
Characteristics of the clusters of changes in lifestyles in confinement. (**A**) Lifestyles: Usual moderate physical activity (PA) and moderate PA in confinement (≥2/week 30-min session), sedentary time in confinement (>8 h/day), poor sleep quality in confinement, exposure to sunlight in confinement (≤1 day/week), usual diet quality (Q1), consumption of supplements in confinement, and smoking. (**B**) Factor scores of patterns of dietary change. (**C**) Sociodemographic characteristics. PA: physical activity; d: day: wk: week; Q1: first quartile of usual diet quality score; inh: inhabitants. ** *p* < 0.01; * *p* < 0.05 χ^2^ test.

Three clusters of change in lifestyles were identified, considering patterns of change in eating habits and change in PA during lockdown. Change in PA was the main predictor. Figure 3 describes the characteristics of these profiles, according to lifestyles in confinement and usual PA (A), patterns of dietary change (B), and sociodemographic characteristics (C). An additional description of the clusters by gender is provided in Supplementary Table S2. One of the clusters grouped together people who were less active than usual during lockdown (C1 Less active) (*n* = 294; 29.5%). Mean factor scores for the rice–pasta–chicken pattern and for the fish pattern were higher in this cluster (Figure 3B). Individuals classified in the less active cluster reported less frequent exposure to sunlight as well. A significantly lower proportion of women and a lower proportion of people aged 18–34 years were in this cluster compared to the other groups identified. A significantly higher proportion of people living alone in confinement, particularly women, was classified in this cluster. The proportion of women who reported being usually more active was significantly higher in this cluster. The proportion of people with poorer diet quality of usual diet was lower in this cluster compared to the other groups.

A second cluster grouped people who reported being more physically active than usual (C2 More active) (*n* = 313; 31.4%). In this profile, mean factor scores for the Healthy Mediterranean-Style dietary pattern of dietary changes were higher. The meat pattern and the dairy pattern scored higher as well. A higher proportion of women and people aged 18–34 years was classified in the more active cluster (Figure 3C). Among women, 52.6% aged 18–34 years were included in C2 more active cluster. A higher proportion of people who practiced three or more 30-min sessions/week of moderate PA was classified in this cluster, but there was also a higher proportion of those who reported more than 8 h of sedentary lifestyle a day and poorer sleep quality in confinement, particularly women.

The third cluster included people who maintained the same level of PA as previously (C3 Active as usual) (*n* = 390; 39.1%). In this profile, the mean factor score of the Unhealthy snack foods and beverage pattern was higher and the mean factor score of the Healthy Mediterranean-Style dietary pattern was lower (Figure 3B). A higher proportion of men and people aged 55 years and over were classified in this cluster (Figure 3C). The proportion of people reporting poorer sleep was significantly higher in this cluster, particularly among men.

In the adjusted model, men were less likely to be classified in the cluster that increased PA in confinement compared to women. People under 55 years were associated with higher odds of being classified in the C2 More active cluster, especially the younger people aged 18–34 years compared to those aged 55 years and over. Participants reporting a lower level of usual PA were more likely to be classified in the cluster that assembled people who reported to be more active during lockdown compared to the cluster grouping together people who reported to be as active as usual during confinement. Exposure to sunlight of ≥10 min one day a week or less was associated with a higher probability of belonging to the less active cluster compared to those with more frequent exposure to sunlight (Table 2).

## 4. Discussion

In this study, different patterns of change in eating habits were identified in adults during lockdown due to COVID-19 in Spain. On the one hand, these patterns show changes toward healthier habits with increased consumption of vegetables, fruits, legumes, and fish, but in other cases, they show changes toward a higher consumption of alcoholic and sugary beverages, and processed products with high fat and salt or sugar content. Furthermore, it was observed that these changes tend to cluster with modifications in PA during lockdown. Cluster analysis identified three clusters defined mainly by change in PA: a group that reported being less active than usual (C1 Less active), a group that reported being more active than usual (C2 More active), and a third group that reported being as active as usual (C3 Active as usual). Age under 55 years was associated with a higher probability of grouping in the more active cluster (C2), while men were associated with a lower probability of classification in this group. People who were usually less active were more likely to classify in the group that increased PA. The group more active during lockdown scored higher in the pattern of dietary changes toward a healthier profile.

Studies carried out in different countries have reported changes in eating habits associated with lockdown regardless of the degree of restrictions imposed. A number of those studies report greater consumption of comfort foods with a high sugar content, such as chocolate and salty snacks [35], which participants attributed to the state of increased anxiety [10]. In this study, we identified an Unhealthy snack foods and beverage pattern of change in eating habits that reflects this trend. Mean factor scores of Unhealthy snack foods and beverage pattern were higher in the less active cluster of change in lifestyles (C1 less active). Moreover, studies report increased overeating during lockdown in people with a high body mass index [13], and negative mood has been associated with increased overeating in confinement [6] as well.

Other authors have reported a higher adherence to the Mediterranean Diet in adults in Spain during confinement [8]. One of the patterns identified in this study shows changes compatible with greater adherence to the Mediterranean diet. Mean factor scores on this pattern were higher in the more active cluster (C2 More active). Studies conducted in other countries report changes consistent with healthier diets in adults during lockdown [36].

The COVID-19 pandemic may be associated to additional negative health impact beyond that produced directly by the viral infection and its consequences. Several systematic reviews analyzing changes in eating habits related to lockdown in different countries conclude that changes leading to increased intake of sugars, fats, and salt are frequent [37]. In a study conducted at the beginning of the COVID-19 pandemic in Spain and Greece, they observed high scores in inappropriate eating behaviors, including overeating in response to negative feelings (external eating behavior) [38].

It is well documented that lower education and socioeconomic level are associated with poorer dietary habits [39], with lower consumption of fresh vegetables and fruits and higher consumption of lower price foods energy dense foods with a high sugar, fat, and salt content. In Spain, greater adherence to the Mediterranean diet has been associated to higher education and socioeconomic level [40]. Several studies document unhealthier dietary changes during lockdown associated to lower socioeconomic level [41]. However, in this study, we did not consider education or income information, and we could not explore the influence of those factors on dietary changes or lifestyle modifications.

On the other hand, studies report increased sedentary lifestyle, screen time, and decreased exposure to sunlight as health-related behaviors modified during lockdown in different countries [12,16,17,35,36]. Other research described an increase in inactivity during lockdown among children [12,33] and adolescents [35] associated with a higher educational level of mothers.

In Spain, outdoor activity outside the home was strictly restricted during lockdown, including for PA. Other research reported less time of PA and increased sedentary lifestyle during lockdown in adults in Spain [14] and in other countries [36]. In countries with less restrictive confinement measures, which allowed going out of the home to practice PA in open air, an increase in PA has been reported, but also increased time dedicated to sedentary activities, with a negative impact on perceived health [16].

In Spain, being physically active at home during lockdown was encouraged [42]. Social media and television channels broadcast daily guided training sessions [43], which were adequate even for people who were not usually involved in active training. Market analysis reported an increase in sales in equipment for practicing exercise at home during the period, such as stationary bicycles, treadmills, weights, and mats [44]. It is possible that these actions have favored that, compared to the Greeks, Spaniards performed more PA and had less inappropriate eating behaviors, despite having faced a more severe COVID-19 pandemic and with stricter confinement measures than in Greece [38]. In line with the above, in our study, we identified a cluster of individuals reporting to be more active than usual in lockdown (C2), including people who usually were not active—more women and people aged 18–54 years. In contrast, subjects who were usually more active reported being less active in confinement.

Some research has shown that the combination of changes in diet, decreased PA, and longer sedentary time during lockdown is associated with poorer quality of sleep [15,38], but this finding is not consistent across studies [45]. In the present study, we observed a higher proportion of people who reported poorer sleep quality in the cluster that maintained their usual activity level during confinement.

Studies in the context of lockdown for the COVID-19 pandemic in different countries report an increase in subjective burden and a decrease in mental and physical wellbeing, which were associated with decreased sleep quality and sleep duration. In these studies, increases in daylight exposure and exercise were associated with less decreased sleep quality and increased sleep duration. The authors speculate that these factors were possibly able to reduce lockdown-induced stress [46]. Furthermore, exposure to natural daylight, obtaining a daily dose of daylight exposure even during a prolonged period of confinement, and exercise are among the strategies suggested to mitigate the adverse effects of the lockdown on sleep quality and health-related behaviors [47].

Sunlight exposure contributes to vitamin D synthesis. In this study, we observed a higher proportion of people who were exposed to sunlight less than one day per week in the cluster that reported less activity during lockdown. Adequate levels of vitamin D have been associated with a lower risk of mortality from chronic diseases [48]. Vitamin D levels have also been associated with outcomes of COVID-19 in hospitalized patients and with testing results, although to date, evidence is limited [49,50]. In this study, 21.3% of participants declared consuming nutritional supplements during lockdown, which included a higher proportion of women and people aged 35–54 years. Although multivitamins were the most widely consumed supplement, 25.8% reported consuming vitamin D supplements and 22% reported consuming vitamin C. In a study conducted in China during confinement, 37.7% of participants reported consuming supplements with vitamin C, probiotics, and other dietary supplements [11].

A number of articles and reviews published during the pandemic highlighted the need to assess the adequacy of nutrition intake and nutritional status and to weigh the convenience of supplementation in specific population groups [22,23].

One of the strengths of this analysis is that it considered the usual diet of participants, the degree of adherence to the dietary guidelines, and the usual physical activity along variations in lifestyles in confinement. In addition, the online form began data collection after the first weeks of confinement, once people adapted to the new situation. To our knowledge, this is the first analysis of patterns of dietary changes and lifestyle variations during confinement for the COVID-19 pandemic, considering sunlight exposure and supplement use. Among the limitations of this cross-sectional study, it is worth mentioning that it was conducted in a convenience sample recruited online, which entails a selection bias. The use of an online survey was necessary during the pandemic, although it may have biased the sample toward those more digitally competent individuals and therefore deviating response rates across societal groups. The online collection of information limits the participation of underprivileged sectors and older people, while it probably eases a higher rate of acceptance by people with greater concern about food, health, and self-care. As reported in other online surveys conducted during the pandemic [8,9,38], in this study, a high proportion of participants were women (70.9%). The lower participation of the male gender in the sample could introduce a gender bias in the results. On the other hand, we did not collect information on the educational or socioeconomic level of participants. Many of the cross-sectional studies conducted during the pandemic aimed to analyze modifications in health-related behaviors [8,9,33,51,52,53] report an over representation of higher educated people, and in many cases, younger age groups are overrepresented as well. In our study, we cannot assess the potential unequal distribution regarding the level of education. However, the sample is evenly distributed by age group, gender distribution across age groups, by region, and by size of locality.

## 5. Conclusions

In conclusion, different patterns of change in eating habits in adults during lockdown for COVID-19 in Spain tend to cluster with modifications in PA. People who were usually less active were more likely to be classified in the cluster that increased PA in confinement. This group scored higher in the Healthier Mediterranean-Style pattern of dietary change. These different patterns of change in lifestyles in confinement suggest the need to tailor support and advice to different population groups. At this time of uncertainty, there is a need to convey clear messages to the population that value the importance of following healthy dietary patterns, such as the Mediterranean diet and lifestyle, adequate levels of PA, outdoors whenever possible, and promoting resilience and emotional balance at the individual and group level.

## Figures and Tables

**Table 1:** Sociodemographic characteristics of participants, general and specific in relation to the lockdown period, by gender.

		Total		Men		Women	
		*n*	%	*n*	%	*n*	%
**Total**		1036	100	301	29.1	735	70.9
Age group	18–34 years	293	28.3	58	19.3	235	32.0
	35–54 years	364	35.1	88	29.2	276	37.6
	55 years and over	379	36.6	155	51.5	224	30.4
Region	North	409	39.5	145	48.2	264	36.0
	Center	295	28.5	75	24.9	220	30.0
	East	135	13.0	31	10.3	104	14.2
	South	197	19.0	50	16.6	147	19.9
Size of locality	<5000 inh.	191	18.4	58	19.3	133	18.1
	5000–19,999 inh.	139	13.4	30	10.0	109	14.7
	20,000–99,999 inh.	178	17.2	61	20.3	117	15.9
	100,000–299,999 inh.	202	19.5	59	19.6	143	19.5
	≥300,000 inh.	326	31.5	93	30.9	233	31.7
Cohabitation during lockdown	Alone	98	9.5	30	10.0	68	9.3
	With a family	614	59.3	164	54.5	450	61.3
	With a partner	261	25.2	90	29.9	171	23.2
	With relatives, friends, or other people	63	6.1	17	5.6	46	6.3
Daylight in the home and outdoor living space during confinement	Windows that allow daylight in	930	89.8	276	91.7	653	88.8
	Small balcony	408	39.4	126	41.9	282	38.4
	Large terrace	457	44.1	119	39.5	337	45.8
	Large outdoor area	240	23.1	70	23.2	169	22.9
	Neither window nor balcony	3	0.3	2	0.7	1	0.1
Smoker	No	879	85.3	262	87.3	616	84.4
	Yes	152	14.7	38	12.7	114	15.6
Usual moderate physical exercise *	Nothing-<1/week	127	13.9	33	12.1	94	14.7
	≥2/week	784	86.1	239	87.9	544	85.3
Usual diet quality score (mean ± SD)		8.79 ± 2.21		8.74 ±1.99		8.82 ± 2.30	

* 30 min-sessions of moderate-vigorous physical activity; inh.: inhabitants; SD: standard deviation.

**Table 2:** Association between cluster of change in physical activity and dietary habits during lockdown, sociodemographic factors, and usual lifestyles.

		C1_Less Active vs. C3 Active as Usual	C2_More Active vs. C3 Active as Usual
		OR (95%CI LL-UL)	OR (95%CI LL-UL)
**Gender**			
	Men	0.77 (0.55–1.07)	0.34 (0.23–0.50)
	Women	1	1
**Age group**			
	18–34 years	0.96 (0.64–1.45)	3.14 (2.11–4.66)
	35–54 years	1.55 (1.09–2.21)	1.73 (1.17–2.57)
	≥55 years	1	1
**Usual moderate physical exercise**			
	<1/week	0.50 (0.29–0.87)	2.12 (1.34–3.35)
	≥2/week	1	1
**Usual diet quality score**			
	Q1	0.80 (0.51–1.25)	1.20 (0.78–1.87)
	Q2–Q3	1.22 (0.82–1.81)	0.98 (0.65–1.48)
	Q4	1	1
**Exposure to sunlight in confinement ≥10 min.**			
	≤1 day/week	1.61 (1.17–2.23)	1.15 (0.82–1.62)
	≥2 day/week	1	1

Multinomial logistic regression analysis, Cluster of change in physical activity and dietary habits as dependent variable with C3 Active as usual, as reference category. 95%CI: 95% confidence interval; LL: lower limit; UL: upper limit.

## Data Availability

The data presented in this study are available on request from the corresponding author.

## Supplementary Materials

The following are available online at https://www.mdpi.com/2072-6643/13/2/300/s1, Table S1: Food groups considered in the food frequency questionnaire and in the questionnaire for self-reported change in consumption, Table S2: Characteristics of clusters of change in physical activity and dietary habits (C) by gender.
