# Supplementary material for: Patterns of Change in Dietary Habits and Physical Activity during Lockdown in Spain Due to the COVID-19 Pandemic
Source: Nutrients. 2021 Jan 21;13(2):300. doi: 10.3390/nu13020300 (PMC7911477; doi:10.3390/nu13020300)
Supplement: Supplementary file 1 [file nutrients-13-00300-s001.pdf]

Table S1. - Food groups considered in the food frequency questionnaire and in the questionnaire for self-reported change in consumption.

**Food groups/ subgroups considered in the food frequency questionnaire**
1. Meat (beef)
2. Meat (pork)
3. Meat (poultry)
4. Processed meats
5. Fish (fresh or frozen)
6. Canned fish
7. Eggs
8. Milk
9. Yoghurt
10. Cheese
11. Legumes
12. Rice, pasta and potatoes
13. Bread
14. Pizza
15. Raw vegetables and salad (potatoes excluded)
16. Cooked vegetables (potatoes excluded)
17. Fresh fruits (juices excluded)
18. Sweet biscuits and bakery products
19. Chocolate, chocolate-based products and confectionery
20. Nuts
21. Salted snacks
22. Sugary drinks
23. Fermented alcoholic drinks (wine, beer, cider)
24. Distilled alcoholic beverages (gin, whisky, vodka, brandy, etc.)

Table S2. - Characteristics of clusters of change in physical activity and dietary habits (C) by gender.

**Variables**		All				Men				Women			
		C1 Less active	C2 More active	C3 As usual	*p*	C1 Less active	C2 More active	C3 As usual	*p*	C1 Less active	C2 More active	C3 As usual	*p*
**Gender**	Men	31.8%	16.6%	51.6%	<0.001								
	Women	28.5%	37.4%	34.0%									
Age groups	18–34 years	20.6%	47.7%	31.7%	<0.001	28.1%	28.1%	43.9%	0.052	18.7%	52.6%	28.7%	<0.001
	35–54 years	34.9%	30.1%	34.9%		38.1%	15.5%	46.4%		34.0%	34.7%	31.3%	
	≥55 years	31.4%	19.6%	49.0%		29.7%	12.8%	57.4%		32.4%	24.3%	43.3%	
Cohabitation	Alone	43.6%	21.3%	35.1%	0.003	40.0%	16.7%	43.3%	ns	45.3%	23.4%	31.3%	0.002
	Family	26.7%	35.6%	37.6%		32.9%	16.5%	50.6%		24.4%	42.7%	32.9%	
	Partner	31.0%	25.3%	43.7%		27.4%	16.7%	56.0%		32.9%	29.8%	37.3%	
	Relatives, friends,	29.0%	29.0%	41.9%		29.4%	17.6%	52.9%		28.9%	33.3%	37.8%	

	other people												
Size of locality	<5,000 inh.<br>5,000-19,999 inh<br>20,000-99,999 inh.<br>100,000-299,999 inh.<br>≥300,000 inh.	25.9%<br>37.5%<br>29.8%<br>29.6%<br>27.9%	27.0%<br>27.9%<br>37.5%<br>31.1%<br>32.4%	47.0%<br>34.6%<br>32.7%<br>39.3%<br>39.7%	ns	28.1%<br>48.3%<br>31.0%<br>33.3%<br>28.4%	10.5%<br>3.4%<br>20.7%<br>21.1%<br>19.3%	61.4%<br>48.3%<br>48.3%<br>45.6%<br>52.3%	ns	25.0%<br>34.6%<br>29.1%<br>28.1%<br>27.7%	34.4%<br>34.6%<br>46.4%<br>35.3%<br>37.5%	40.6%<br>30.8%<br>24.5%<br>36.7%<br>34.8%	ns
Smoker	Yes	28.4%	28.4%	43.2%	ns	27.8%	5.6%	66.7%	ns	28.6%	35.7%	35.7%	ns
Smoking in confinement	More	31.3%	37.5%	31.3%	ns	62.5%	0.0%	37.5%	ns	25.0%	45.0%	30.0%	ns
Supplements in confinement	Yes	33.3%	25.2%	41.4%	ns	32.7%	18.4%	49.0%	ns	33.5%	27.3%	39.1%	0.011
Exposure to sunlight in confinement	Exposure ≥10min ≤1day/week	35.2%	29.9%	34.9%	0.02	38.0%	20.0%	42.0%	ns	33.9%	34.4%	31.7%	ns
Sleep in confinement	Poor quality	30.7%	32.3%	37.0%	<0.001	40.6%	15.9%	43.5%	<0.001	28.0%	36.8%	35.2%	0.018
Sedentary behavior in confinement	>8h/day	29.8%	36.5%	33.7%	0.020	29.8%	17.5%	52.6%	ns	29.7%	42.1%	28.2%	0.026
Usual moderate PA	30min sessions ≥2days/week	36.2%	25.5%	38.3%	<0.001	36.8%	14.5%	48.7%	<0.001	35.9%	30.3%	33.8%	<0.001
Moderate PA in confinement	30min sessions ≥2days/week	17.6%	41.1%	41.3%	<0.001	22.0%	22.5%	55.5%	<0.001	15.9%	48.6%	35.5%	<0.001
Quality of usual diet	Q1	23.1%	36.7%	40.3%	0.021	21.6%	20.5%	58.0%	ns	23.6%	43.2%	33.2%	ns

C: cluster of change in physical activity and dietary habits. PA: physical activity. Q1: first quartile of usual diet quality score; inh: inhabitants. χ^2 test.
